# Some chaotic properties of fuzzified dynamical systems

**DOI:** 10.1186/s40064-016-2297-z

**Published:** 2016-05-17

**Authors:** Cuina Ma, Peiyong Zhu, Tianxiu Lu

**Affiliations:** School of Mathematical Sciences, University of Electronic Science and Technology of China, 611731 Chengdu, People‘s Republic of China; Department of Mathematics, Sichuan University of Science and Engineering, 643000 Zigong, People‘s Republic of China

**Keywords:** *g*-Fuzzification, Turbulent, Erratic, *λ*-Expansive

## Abstract

Let *X* be a compact metric space and $f:X\longrightarrow X$ a continuous map. Considering the space ${{\mathbb {F}}}(X)$ of all nonempty fuzzy sets on *X* endowed with the levelwise topology, we proved that its *g*-fuzzification is turbulent or erratic if the given system *f* is turbulent or erratic correspondingly and *f* is $\lambda$-expansive if and only if its *g*-fuzzification is $\lambda$-expansive.

## Background

Let *X* be a compact metric space and *f* be a continuous self-map. We denote by ${{\mathbb {F}}}(X)$ all nonempty fuzzy sets on *X* endowed with the levelwise topology and we define $\widehat{f}$ as the Zadeh’s extension of the map *f*:

$$\begin{aligned} \widehat{f}(u(x))=\sup \limits _{y\in f^{-1}(x)}(u(y)), \quad u\in {{\mathbb {F}}}(X). \end{aligned}$$

With the complexity of situations rising, the zadeh’s extension has failed to be an accurate description for discrete fuzzy systems affected by the uncertainty. The time evolution of a chosen initial state is very important. We take the fuzzy set “short people” for example. “short people” in ancient time are not defined as short at present since the average height of people is increasing. It’s not difficult to find some other examples to figure out that the zadeh’s extension can’t reflect the complex systems accurately. A very important generalization of the concept of zadeh’s extension is *g*-fuzzification introduced in Kupka (2011a). It can be used for changing relevant membership grades and developed to describe the complex fuzzy systems in a more efficient way. The map $\widehat{f}_{g}: {{\mathbb {F}}}(X)\longrightarrow {{\mathbb {F}}}(X)$ is called *g*-fuzzification of *f* and ${{\mathbb {F}}}(X)$ denotes all nonempty and fuzzy compact subsets of *X*. Over the last ten or so years, since many research works have been devoted to the chaotic behaviors of the fuzzified dynamical systems, such as, topological entropy, Devaney chaos, Li-Yorke chaos and its connections with erratic functions which has been done by Cánovas and Kupka (2011), Kupka (2011b), Diamond (1994), Diamond and Kloeden (1994), Diamond and Pokrovskii (1994). Recently, J.Kupka have studied various chaotic behaviors (Li-Yorke chaos, $\omega$-chaos, distributional chaos, topological chaos etc.) between a given dynamical system (*X*, *f*) and its *g*-fuzzification. See more details in Kupka (2014). But it remains to be asked to study the other chaotic properties on *g*-fuzzification. Then the turbulence and erratic properties between a crisp dynamical system and its *g*-fuzzification are taken into our considerations in this paper.

On the other hand, the $\lambda$-expansive property in fuzzy systems is explored in this paper. Thus, the left work is to demonstrate the $\lambda$-expansiveness relationship between $\widehat{f}_{g}$ and *f*. Meanwhile, it is not difficult to see that the $\lambda$-expansive property can exhibit the sensitivity of $\widehat{f}_{g}$.

## Preliminaries

### Metric space of fuzzy sets

Let (*X*, *d*) be a compact metric space and *C*(*X*) denote all continuous functions. $f\in C(X)$ is a self-map. ${{\mathcal {K}}}(X)$ denotes all nonempty and compact subsets of *X* with the Hausdorff metric $d_{H}$ defined by

$$\begin{aligned} d_{H}(A, B)=\max \left\{ \sup _{x\in A}\inf _{y\in B}d(x, y), \sup _{y\in B}\inf _{x\in A}d(x, y)\right\} \end{aligned}$$

for any $A, B\in {{\mathcal {K}}}$. $\bar{f}: {{\mathcal {K}}}(X)\longrightarrow {{\mathcal {K}}}$ is defined as $\bar{f}(A)=f(A)$ for any $A\in {{\mathcal {K}}}$.

A fuzzy set *u* on the space *X* is a function $u: X\longrightarrow I$ where $I=[0,1]$. For any $\alpha \in (0,1], [u]_{\alpha }=\{x\in X\mid u(x)\ge \alpha \}$ is called the $\alpha$-level of *u* and $[u]_{0}=\{x\in X\mid u(x)\ge 0 \}$ is the support of *u* (shortly: supp(u)) (Román-Flores et al. 2011).

Throughout this paper, ${{\mathbb {F}}}(X)$ is defined as all upper semi-continuous fuzzy sets and equipped by the following metric:

$$\begin{aligned} d_{\infty }(u,v)= \sup \limits _{\alpha \in (0,1]} d_{H}([u]_{\alpha },[v]_{\alpha }). \end{aligned}$$

Let

$$\begin{aligned} {{\mathbb {F}}}^{\lambda }(X)=\{ A\in {{\mathbb {F}}}(X) \mid A(x)\ge \lambda \} \end{aligned}$$

and ${{\mathbb {F}}}^{1}(X)$ is denoted as the system of all normal fuzzy sets on X.

### *g*-fuzzifications

Let $D_{m}(I)$ be the set of all nondecreasing right-continuous functions $g: I\longrightarrow I, I=[0,1]$. If $x=0$ and $x=1$, $g(x)=x$. A map $\widehat{f}_{g}$ denoted by

$$\begin{aligned} \widehat{f}_{g}(x)=\sup \limits _{y\in f^{-1}(x)}\{g(u(y))\} \ \hbox { for } \ \hbox { any }\ u\in {{\mathbb {F}}}(X),\ x\in X \end{aligned}$$

is defined as a *g*-fuzzification. Especially, it is the Zadeh’s extension when the function $g(x)=1$. Also, a definition of $\alpha$-cut $[A]^{g}_{\alpha }$ is presented by $[A]^{g}_{\alpha }=\{x\in [A]_{0}\mid g( A(x))\ge \alpha \}$. The following equation in Kupka (2011a) is indispensable.

$$\begin{aligned} f([A]_{\alpha }^{g})=[\widehat{f}_{g}(A)]_{\alpha },\quad \forall A\in {{\mathbb {F}}}(X). \end{aligned}$$

#### **Lemma 1**

Kupka (2014): *Let*$f\in C(X)$*and*$g\in D_{m}(I)$. *For any*$\alpha \in (0,1]$*and nonempty fuzzy set*$A\in {{\mathbb {F}}}(X)$, *if*$[A]^{g}_{\alpha }\ne \emptyset$*then there is*$c\in (0,1]$*such that*$[A]^{g}_{\alpha }=[A]_{c}$.

To explore some properties of *g*-fuzzification, for any $U\subset X$, we define

$$\begin{aligned} e_{\alpha }(U)= \{\hbox {B }\in {{\mathbb {F}}}(X)\mid [B]_{\alpha }\ne \emptyset \hbox { and } [B]_{\alpha }\subseteq U \} \end{aligned}$$

and

$$\begin{aligned} \vartheta (U)= e_{1}(U)\cap e(U),\\ e(U)=\{A\in {{\mathbb {F}}}(X)\mid \hbox { supp }(A)\subseteq U \}. \end{aligned}$$

Clearly, $e(U)\ne \emptyset$ if and only if $U \ne \emptyset$. Moreover, the following lemma is essential in the paper.

#### **Lemma 2**

Kupka (2014): *LetU*, *Vbe two subsets of*$X, f\in C(X)$*and*$g\in D_{m}(I)$. *Then:*

1. $$e(U \cap V)=e(U)\cap e(V).$$
2. $$\widehat{f}_{g}(e(U))\subseteq e(f(U)).$$
3. $$\widehat{f}_{g}(e(U))= e(f(U))\ whenever\ U\ is\ closed.$$

Likewise, $\vartheta (U)$ has the same properties compared with *e*(*U*).

#### **Lemma 3**

Kupka (2014): *LetU*, *Vbe two subsets of*$X, f\in C(X)$*and*$g\in D_{m}(I)$. *Then:*

1. $$\vartheta (U \cap V)=\vartheta (U)\cap \vartheta (V)$$.
2. $$\widehat{f}_{g}(\vartheta (U))\subseteq \vartheta (f(U))$$.
3. $$\widehat{f}_{g}(\vartheta (U))= \vartheta (f(U))\ whenever\ U\ is\ closed$$.

### Turbulence, erratic property, Block and Coppel chaos and $\lambda$-expansiveness

Before we present the elegant results, we need to introduce some basic definitions of chaotic behavior explored in this paper.

#### **Definition 1**

Román-Flores et al. (2011): Let $f: X\longrightarrow X$ be a continuous function. We say that *f* is a turbulent function if there are disjoint nonempty closed subsets $J,\ K$ of *X* such that:

$$\begin{aligned} J\cup K\subseteq f(J)\cap f(K). \end{aligned}$$

#### **Definition 2**

Román-Flores et al. (2011): A map $f\in C(X)$ is erratic if there exists a nonempty closed set $A\subseteq X$ satisfying the following two conditions:

1. $$A \cap f(A) = \emptyset$$.
2. $$A \cup f(A) \subseteq f^{2}(A)$$.

#### **Definition 3**

Román-Flores et al. (2011): Let $f: X\longrightarrow X$ be a continuous function.We say that *f* is chaotic in the sense of Block and Coppel (in short: B-C chaos) if and only if its iterates is turbulent, i.e., there exists $n\ge 1$ and two disjoint nonempty compact subsets *J*, *K* of *X* such that

$$\begin{aligned} J\cup K\subseteq f^{n}(J)\cap f^{n}(K). \end{aligned}$$

It should be remarkable that the erratic property is stronger than B-C chaos.

#### **Definition 4**

Let $f: X\longrightarrow X$ be a function. We claim that *f* is expansive if and only if there exists a real constant $\lambda >1$ such that

$$\begin{aligned} d(f(x),f(y))\geqslant \lambda d(x,y), \ \forall x, y \in X. \end{aligned}$$

In this case we claim that *f* is $\lambda$-expansive Román-Flores and Chalco-Cano (2005).

## Main results

To prove the turbulent and erratic properties, firstly, a theorem on *e*(*U*) is given here.

### **Theorem 1**

*LetUbe closed subset ofXand*$e(U)=\{\hbox { B }\in {{\mathbb {F}}}(X)\mid \hbox { supp}(\hbox {B})\subseteq U\}, f\in C(X)$*and*$g\in D_{m}(I)$. *Then,*

$$\begin{aligned} \widehat{f_{g}^{n}} (e(U))= \widehat{f}_{g}^{n}(e(U)). \end{aligned}$$

### *Proof*

It can be proved by the mathematical induction.

When $n=1$, Left = Right = $\widehat{f}_{g}(e(U))$. Clearly, the theorem holds.

Assume that the theorem is true for $n=k$, i.e.,

$$\begin{aligned} \widehat{f_{g}^{k}} (e(U))= \widehat{f}_{g}^{k}(e(U)). \end{aligned}$$

By the Lemma 2 in Kupka (2014), we have

$$\begin{aligned} \widehat{f^{k}_{g}} (e(U))=e(f^{k}(U)). \end{aligned}$$

When $$n=k+1$$,

$$\begin{aligned} \widehat{f^{k+1}_{n}} (e(U))&=e(f_{n}^{k+1}(U))=e(f_{n}\circ (f_{n}^{k}(U)))\\&= \widehat{f}_{n}(e(f_{n}^{k}(U)))\\&= \widehat{f}_{n}\circ \widehat{f}_{n}^{k} (e(U))\\&= \widehat{f}_{n}^{k+1} (e(U)). \end{aligned}$$

Consequently, the statement is proved completely. $\square$

Likewise, we can achieve the similar result on $\vartheta (U)$.

### **Theorem 2**

*LetUbe closed subset ofXand*$\vartheta (U)=e_{1}(U)\cap e(U)$. $f\in C(X)$*and*$g\in D_{m}(I)$. *Then,*

$$\begin{aligned} \widehat{f^{n}_{g}} (\vartheta (U))= \widehat{f}_{g}^{n}(\vartheta (U)). \end{aligned}$$

Here, we make a point that $\forall \alpha \in (0,1]$,

$$\begin{aligned} \widehat{f^{n}_{g}} (e_{\alpha }(U))\ne \widehat{f}_{g}^{n}(e_{\alpha }(U)). \end{aligned}$$

See Lan and Mu (2014) for more details.

Román-Flores et al. (2011) explored the dynamics of $\widehat{f}$ and *f*. These conclusions can be listed as follows:

$$\begin{aligned}&f \hbox { is turbulent (erratic) } \Rightarrow \widehat{f} \hbox { is turbulent (erratic) }\\&\widehat{f} \hbox { is turbulent (erratic) } \nRightarrow f \hbox { is turbulent (erratic)}. \end{aligned}$$

More precisely, Román-Flores et al. (2011) present some examples in order to show the irreversible links between *f* and $\widehat{f}$. We generalizes the results with $\widehat{f_{g}}$. As the Zadeh’s extension is a spacial case of the g-fuzzification, we can make a conclusion:

$$\begin{aligned} \widehat{f}_{g} \hbox { is turbulent (erratic) }&\nRightarrow&f \hbox { is turbulent (erratic)}. \end{aligned}$$

The left work is to prove the following implication:

$$\begin{aligned}&f \hbox { is turbulent (erratic) } \Rightarrow \widehat{f}_{g} \hbox { is turbulent (erratic)}. \end{aligned}$$

### **Theorem 3**

*If*$f\in C(X), f: X\longrightarrow X$*is a turbulent function, then*$\widehat{f}_{g}: {{\mathbb {F}}}(X)\longrightarrow {{\mathbb {F}}}(X)$*is a turbulent function*.

### *Proof*

Since *f* is turbulent, there exists nonempty and closed $U, V\subseteq X$ and $U\cap V=\emptyset$ such that $U\cup V \subseteq f(U)\cap f(V)$.

By the definition of *e*(*U*), clearly, *e*(*U*) and *e*(*V*) are two disjoint nonempty closed subsets of ${{\mathbb {F}}}(X)$. Applying the Lemma 2, we have

$$\begin{aligned} e(U)\cup e(V)\subseteq e(U\cup V)&\subseteq e(f(U)\cap f(V))\\&= e(f(U))\cap e(f(V))\\&= \widehat{f}_{g}(e(U))\cap \widehat{f}_{g}(e(V)). \end{aligned}$$

Finally, we can conclude that $\widehat{f}_{g}$ is a turbulent function. $\square$

### **Theorem 4**

*Let*$f\in C(X)$*be erratic, then,*$\widehat{f}_{g}$*is a erratic function*.

### *Proof*

Since *f* is erratic, there exists a nonempty closed subset $U\subseteq X$ such that $U\cap f(U)=\emptyset$ and $U\cup f(U)\subseteq f^{2}(U)$. Thus, *e*(*U*) is nonempty closed subset of ${{\mathbb {F}}}(X)$ and

$$\begin{aligned} e(U)\cap \widehat{f}_{g}(e(U))&= e(U)\cap e(f(U))\\&= e((U) \cap f(U))\\&= e(\emptyset )=\emptyset . \end{aligned}$$

On the other hand, using the Theorem 1 and the Lemma 2, we have

$$\begin{aligned} e(U)\cup \widehat{f}_{g}(e(U))&= e(U)\cup e(f(U))\\&\subseteq e(U\cup f(U))\\&\subseteq e(f^{2}(U))\\&= \widehat{f}_{g}^{2}(e(U)). \end{aligned}$$

Consequently, $\widehat{f}_{g}$ is erratic. $\square$

### **Corollary 1**

*Let*$f\in C(X)$*be erratic, then*, $\widehat{f}_{g}$*is a B-C chaos function*.

### *Remark 1*

Because $\vartheta (U)$ has similar properties to the *e*(*U*), it can be verified with $\vartheta (U)$ that the statements are true. Comparing Theorem 3 with Theorem 4, the fuzzy set containing *e*(*U*) at least is perfect for the two theorems to make sense.

Next, we shall discuss the $\lambda$-expansive property of $\widehat{f}_{g}$.

### **Theorem 5**

*Let*$f: X\longrightarrow X$*be a continuous function and*$g\in D_{m}(I)$. *Thenfis*$\lambda$-*expansive if and only if*$\widehat{f}_{g}$*is*$\lambda$-*expansive*.

### *Proof*

($\Rightarrow$) Since *f* is $\lambda$-expansive, for any $u,v\in {{\mathbb {F}}}(X)$ and $\alpha \in (0,1]$, it follows that

$$\begin{aligned}&\sup d_{H}(f([u]_{\alpha }),f([v]_{\alpha }))\\&\quad =\max \limits _{\alpha \in (0,1]}\{\sup \limits _{x\in [u]_{\alpha }}\inf \limits _{y\in [v]_{\alpha }}d(f(x),f(y)),\\&\qquad \sup \limits _{y\in [v]_{\alpha }}\inf \limits _{x\in [u]_{\alpha }}d(f(x),f(y))\}\\&\quad \ge \max \limits _{\alpha \in (0,1]}\{\sup \limits _{x\in [u]_{\alpha }}\inf \limits _{y\in [v]_{\alpha }}\lambda d(x,y),\sup \limits _{y\in [v]_{\alpha }}\inf \limits _{x\in [u]_{\alpha }}\lambda d(x,y)\}\\&\quad =\lambda \max \limits _{\alpha \in (0,1]}\{\sup \limits _{x\in [u]_{\alpha }} \inf \limits _{y\in [v]_{\alpha }}d(x,y),\sup \limits _{y\in [v]_{\alpha }}\inf \limits _{x\in [u]_{\alpha }}d(x,y)\}\\&\quad =\lambda \sup d_{H}([u]_{\alpha },[v]_{\alpha })\\&\quad =\lambda d_{\infty }(u,v). \end{aligned}$$

Applying $f([A]_{\alpha }^{g})=[\widehat{f}_{g}(A)]_{\alpha }$, it follows that

$$\begin{aligned} d_{\infty }(\widehat{f}_{g}(u), \widehat{f}_{g}(v))&= \sup d_{H}([\widehat{f}_{g}(u)]_{\alpha }, [\widehat{f}_{g}(v)]_{\alpha } )\\&= \sup d_{H}(f([u]^{g}_{\alpha }),f([v]^{g}_{\alpha })). \end{aligned}$$

By the Lemma 1, there is $c\in (0,1]$ such that

$$\begin{aligned} \sup d_{H}(f([u]^{g}_{\alpha }),f([v]^{g}_{\alpha }))&= \sup d_{H}(f([u]_{c}),f([v]_{c}))\\&\ge \lambda d_{\infty }(u,v), \end{aligned}$$

which implies $\widehat{f}_{g}$ is $\lambda$-expansive.

($\Leftarrow$) Based on the two following equations:

$$\begin{aligned} d_{\infty }(\chi _{\{x\}}, \chi _{\{y\}})=d(x,y) \end{aligned}$$

and

$$\begin{aligned} d_{\infty }(\widehat{f}_{g}(\chi _{\{x\}}),\widehat{f}_{g}(\chi _{\{y\}}))=d(f(x), f(y)), \end{aligned}$$

it is obvious that *f* is $\lambda$-expansive. $\square$

### **Corollary 2**

*If*$\widehat{f}_{g}$*is*$\lambda$-*expansive*, $\lambda >1$, *then*$\widehat{f}_{g}$*is sensitively dependent*.

### *Proof*

Applying the method of induction, it is obvious that

$$\begin{aligned} d_{\infty }(\widehat{f}_{g}^{n}(u), \widehat{f}_{g}^{n}(v))\ \ge \ \lambda ^{n}\ d_{\infty }(u,v)\rightarrow \infty ,\ as\ n\rightarrow \infty . \end{aligned}$$

Thus, $\widehat{f}_{g}$ is sensitively dependent. $\square$

### **Corollary 3**

*Iffis*$\lambda$-*expansive, then*$\widehat{f}_{g}$*is sensitively dependent*.

## Conclusions

In this paper, exploiting the turbulent and erratic properties, we develop the ideas of Román-Flores et al. (2011) and present some properties of *e*(*U*) and $\vartheta (U)$ for $\widehat{f}_{g}$ , which can be applied to the proof of Theorem 3 and Theorem 4. Moreover, the $\lambda$-expansive property between *f* and $\widehat{f}_{g}$ is studied and exhibit the sensitivity. Inducing sensitivity on fuzzy systems contains asymptotic sensitive, Li-Yorke sensitive and spatial-temporal sensitive etc, which will be further investigated and solved in a later work.
